# Increased Cardiopulmonary Fitness Is Associated with a Greater Reduction in Depression among People Who Underwent Bariatric Surgery

**DOI:** 10.3390/ijerph18052508

**Published:** 2021-03-03

**Authors:** Tomas Vetrovsky, Tereza Fortova, Elena Conesa-Ros, Michal Steffl, Jana Heczkova, Jan Belohlavek, Javier Courel-Ibáñez

**Affiliations:** 1 Institute of Nursing Theory and Practice, First Faculty of Medicine, Charles University, 128 00 Prague, Czech Republic; tomas.vetrovsky@gmail.com (T.V.); t.schauerova@gmail.com (T.F.); jana.heczkova@lf1.cuni.cz (J.H.); 2 Faculty of Physical Education and Sport, Charles University, 162 52 Prague, Czech Republic; Steffl@ftvs.cuni.cz; 3 Hospital Jablonec nad Nisou, 466 01 Jablonec nad Nisou, Czech Republic; 4 Human Performance and Sports Science Laboratory, Faculty of Sport Sciences, University of Murcia, 30720 Murcia, Spain; econesaros@um.es; 5 2nd Department of Internal Medicine, Cardiovascular Medicine, General University Hospital, 128 08 Prague, Czech Republic; jan.belohlavek@vfn.cz; 6 1st Faculty of Medicine, Charles University in Prague, 128 00 Prague, Czech Republic

**Keywords:** gastric bypass, obesity, weight loss, mental health, comorbidity

## Abstract

The aim of this study was to determine the effect of changes in cardiopulmonary fitness on the mental health of patients with severe obesity who underwent gastric bypass surgery (prior to and 1, 3, and 6 months after surgery). Study participants were recruited from among patients of a regional hospital in Czechia who underwent gastric bypass surgery between April 2018 and October 2019. They were eligible if they (a) were between 18 and 65 years old, (b) provided written informed consent, and (c) were able to walk independently. Twenty-six patients (age 45.4 ± 9.0 years, body mass index 45.1 ± 7.4 kg·m^−2^, body fat 43.8 ± 4.8%) were included in the analysis. The key finding revealed that the greater the increase in cardiopulmonary fitness (i.e., longer distance walked in the six-minute walk test, 6MWT), the better the improvement in depression score among patients who underwent bariatric surgery. In particular, increments of 10 m in the 6MWT lead to the improvement of 0.5 points on the depression subscale of the Hospital Anxiety and Depression Scale (HADS) questionnaire. As the main implication, these results suggest that patients should participate in exercise training programs to increase their fitness status for optimal physical and mental outcomes of bariatric surgery.

## 1. Introduction

Obesity continues to be a major public health problem with increasingly devastating social, economic, and health consequences [1]. High body-mass index (BMI ≥ 30 kg·m^−2^) increases the risk of comorbidities and chronic diseases, including cardiovascular diseases, diabetes mellitus [2], cancer [3], kidney diseases [4], and musculoskeletal disorders [5], as well as mental and emotional disorders such as depression [6]. With the rapid increase in the prevalence and burden of obesity worldwide, it is urgent to implement efficient and sustainable solutions [7].

Bariatric surgery is effective for weight loss in patients with severe obesity, namely, those with BMI ≥ 40 kg·m^−2^ or BMI ≥ 35 kg·m^−2^ with major medical comorbidities [8,9,10,11]. Despite advancements in medicine and technology, common bariatric surgeries are invasive and costly interventions that carry risks and side effects that require long-term skilled aftercare and may lead to revisional procedures [12]. Patients who seek weight loss surgery are primarily motivated by improving their current medical condition but endorse psychological and quality of life factors as important in their decision [13]. Compared with non-surgical treatments, bariatric surgery leads to greater weight loss (~20 to ~30 kg) [11] with proven long-term effectiveness to sustain most of the weight loss and to control comorbidities [8,14].

Severe obesity often leads to clinical depression, which is present in 40% of individuals selected to undergo bariatric surgery [6,15]. Certainly, bariatric surgery is associated with reductions in anxiety and depressive symptoms [16]. However, preoperative psychological disorders are often maintained and even worsen, although a successful surgical control of obesity was achieved [17,18,19,20,21]. Furthermore, pre-operative depressive and anxiety disorders attenuate post-surgical improvements by means of losing significantly less weight after surgery [15] and promoting greater weight regain [22]. Thus, developing strategies to mitigate depression and enhance mental health in the long term after bariatric surgery requires clinical attention.

There is mounting evidence that increasing cardiopulmonary fitness pre- and postoperative improves the surgery process by greater post-surgery weight loss, improved body composition, and enhanced fitness following surgery [23,24,25,26,27,28,29,30,31]. Conversely, lower levels of cardiopulmonary fitness have been associated with suboptimal weight loss and weight regain post-surgery [32]. In addition to physical benefits, increments in postoperative physical activity positively contribute to better mental health and wellbeing by lowering eating-disorder psychopathology, eating frequency, and shape/weight dissatisfaction [33]. This is relevant, since cognitive–behavioral therapies and psychosocial interventions have a limited impact on eating and lifestyle behaviors [34]. Thus, increased cardiopulmonary fitness may have an important role in physical and mental health through physiological and psychological pathways [33]. However, whereas the inclusion of physical activity and exercise training in the clinical follow-up trajectory for better physical outcomes seems clear [31], evidence regarding the benefits of improved cardiopulmonary fitness for the mental health and wellbeing of post-operative patients is limited. Recent approaches suggest that enrollment in post-operative physical exercise regimes may reduce depression and anxiety symptoms, but not reaching significant results [35,36]. However, the fact that increments in fitness could motivate extra changes towards positive mental health and wellbeing requires further examination.

The aim of this study was to determine the effect of changes in cardiopulmonary fitness on changes in mental health, fatigue, and health-related quality of life of patients with severe obesity who underwent gastric bypass surgery.

## 2. Materials and Methods

### 2.1. Study Design and Population

This prospective observational study assessed cardiopulmonary fitness, physical activity, mental health, fatigue, and health-related quality of life prior to and 1, 3, and 6 months after bariatric surgery. The study was reviewed and approved by the ethics committee of the Hospital Jablonec nad Nisou (2018-04-16).

Study participants were recruited from among the patients undergoing gastric bypass surgery between April 2018 and October 2019 in a regional hospital that performs approximately 100 gastric bypass surgeries every year. The patients were eligible if they (a) were between 18 and 65 years old, (b) provided written informed consent, and (c) were able to walk independently. The patients were individually approached by one of the researchers (T.F.) who served as a nurse in the hospital, but they were not motivated to participate in this study in any way. In total, 40 patients were addressed; of those, 3 refused to participate, 1 was later found to be ineligible (wheelchair user), 7 did not adhere to the study procedures, 1 did not undergo surgery, and 2 had to undergo reoperation. The remaining 26 patients were included in the analysis.

Patients were encouraged to maintain an active lifestyle and reduce sedentary activities, but they did not receive specific guidelines.

### 2.2. Measures

All measures were assessed during patients’ regular visits to the hospital. Anthropometric measures and patient-reported measures of mental health, fatigue, and health-related quality of life were assessed prior to surgery (T0) and at 1, 3, and 6 months (T1, T3, and T6, respectively) after the surgery. However, measures of cardiopulmonary fitness and habitual physical activity were only assessed prior to surgery (T0) and at three months after the surgery (T3) due to practical constraints.

#### 2.2.1. Cardiopulmonary Fitness

The six-minute walk test (6MWT) is a practical, simple test of cardiopulmonary fitness that measures the distance that a patient can quickly walk on a flat, hard surface in a period of 6 min. The test was performed on a 30 m indoor hallway course with a controlled environment. Patients were instructed to walk back and forth in the corridor with the goal to walk as far as possible for 6 min, but they were not allowed to run. Only the standardized phrases for encouragement were used during the test [37]. The 6MWT has shown good reproducibility and validity in subjects with obesity [38], and it is commonly used to evaluate intervention success beyond kilogram weight loss in obese subjects [39]. Reference equations specific for the obese population have been established that can be used as a realistic benchmark in the rehabilitation setting to assess functional capacity, plan exercise intensity, and monitor changes over time [40].

#### 2.2.2. Habitual Physical Activity

The daily number of steps measured by the accelerometer was used to assess habitual physical activity of the patients. The patients were fitted with an Actigraph wGT3X−BT accelerometer (ActiGraph, Pensacola, FL, USA) and were requested to wear it for seven full days during waking hours. The accelerometer was attached to the right hip using an elastic band provided by the manufacturer. Participants were instructed to remove it only when sleeping at night and for water-based activities. Accelerometer data were processed using ActiLife software (version 6.13.3). Non-wear time was detected using the Choi et al. algorithm [41], and only valid days, i.e., those with the minimum wear time of 480 min per day, were included in the analysis. At least three valid days at both T0 and T3 assessments were required for the analysis [42].

#### 2.2.3. Anthropometric Measures

Bodyweight and body fat percentage were determined by bioelectrical impedance analysis using the Tanita MC−780 multi-frequency segmental Body Composition Analyser. Height was measured using a calibrated stadiometer. BMI was calculated by dividing the body weight (kg) by the square of the height (m^2^).

#### 2.2.4. Mental Health

Anxiety and depression subscales of the Hospital Anxiety and Depression Scale (HADS) were used to assess patients’ mental health. The HADS is a 14-item questionnaire consisting of depression and anxiety subscales. The items are graded on a four-point Likert scale from 0–3, with the total score for each subscale ranging from 0–21, where lower scores represent better mental health. A cut-off score of 8 points on both subscales has been found to provide an optimal balance between sensitivity and specificity to detect suspicious clinical cases of depression and anxiety [43]. The HADS has been shown to be valid for detecting mood disorders among the obese and has demonstrated good responsiveness to change in patients operated for morbid obesity [44].

#### 2.2.5. Fatigue

For the assessment of fatigue, the Global Fatigue Index (GFI) was calculated using the Multidimensional Assessment of Fatigue (MAF) questionnaire. The MAF questionnaire consists of 15 items in five separate dimensions (degree, severity, distress, impact on activities of daily living, timing) that generate a single score, the GFI. Each item employs a 10-point numerical rating scale with the total score for GFI ranging from 1–50, where a higher score indicates more severe fatigue, fatigue distress, or impact on activities of daily living. The questionnaire has been originally developed for use with adults who have rheumatoid arthritis [45], but it has been successfully applied to various populations, including obese adults [46,47].

#### 2.2.6. Health-Related Quality of Life

The SF–36 questionnaire is a validated measure of health-related quality of life that consists of 36 questions divided into eight individually analyzed dimensions: (1) limitations in physical activities because of health problems (physical functioning); (2) limitations in usual role activities because of physical health problems (role—physical); (3) limitations in social activities because of physical or emotional problems (social functioning); (4) bodily pain; (5) general mental health; (6) limitations in usual role activities because of emotional problems (role—emotional); (7) vitality; (8) general health perceptions. Each dimension is scored on a 0–100 scale, with higher scores representing better self-reported health. Two summary scores (physical component and mental component) can be calculated from SF–36; however, in the present study, we only analyzed the eight individual dimensions [48].

### 2.3. Statistical Analysis

Means and SDs were calculated for all measures at all time points. Mean differences and their two-sided 95% confidence intervals were calculated for changes from T0 to T1, T3, and T6, respectively.

To examine the effect of change in cardiopulmonary fitness on mental health, fatigue, and quality of life, we conducted a series of linear models. For each outcome variable (anxiety and depression subscales of HADS, GFI, eight dimensions of SF–36), we modeled its change from T0 to T3 and from T0 to T6 as a function of change in 6MWT from T0 to T3 (Model 1). Furthermore, to control for confounders, we also included patients’ age, sex, change in weight, change in the daily number of steps, and presence of comorbidities as covariates in the models (Model 2). The residual plots of all models were visually inspected to verify the assumptions of linearity, homoscedasticity, and normality.

The alpha level of significance was set at 0.05 for each family of hypotheses: mental health, fatigue, and quality of life. We used Bonferroni correction to control for family-wise error within these families. As each family was composed of a different number of hypotheses, the *p*-value was considered to be significant at different alpha levels: *p* < 0.0125 for mental health (4 hypotheses: 2 variables at 2 time points), *p* < 0.025 for fatigue (2 hypotheses: 1 variable at 2 time points), and *p* < 0.003125 for quality of life (16 hypotheses: 8 variables at 2 time points).

All analyses were performed using the statistical package R (version 3.5.2; The R Foundation for Statistical Computing, Vienna, Austria).

## 3. Results

### 3.1. Subjects

The baseline characteristics of 26 patients included in the analysis are depicted in Table 1.

### 3.2. Changes after Surgery

Following the surgery, body weight, BMI, and body fat percentage progressively improved, with the greatest improvements observed at six months after surgery. Mental health and fatigue improved at all time points, and quality of life improved at three and six months after surgery (Table 2). Cardiopulmonary fitness and habitual physical activity increased from baseline assessment prior to surgery to three months after surgery by 36 m and 1260 steps/day, respectively.

### 3.3. The Effect of Changes in Cardiopulmonary Fitness

Improvements in the depression subscale of HADS were significantly affected by changes in cardiopulmonary fitness operationalized as distance walked during the 6MWT. For every 10 m of increase in 6MWT distance, there was an improvement of 0.5 points on the depression subscale (range 0 to 21). This effect was observed at both three and six months and remained significant after controlling for confounders. Improvements in GFI, anxiety subscale of HADS, and individual dimensions of SF–36 were not significantly affected by changes in cardiopulmonary fitness Table 3.

## 4. Discussion

This study examined the relationship between changes in cardiopulmonary fitness and mental health prior to and 1, 3, and 6 months after gastric bypass surgery. The key finding revealed that the greater the increase in cardiopulmonary fitness (i.e., longer distance walked in 6MWT), the better the improvement in depression score among patients who underwent bariatric surgery. In particular, increments of 10 m in the 6MWT lead to the improvement of 0.5 points on the depression subscale of the HADS questionnaire. According to our findings, the observed increase of 36 m in 6MWT at 3 months post-surgery translates to a significant decrease of 1.8 points on the 21-point depression scale. As the minimal clinically important difference for depression score has been triangulated to be between 1.4 and 1.7 [49,50], our finding represents a noticeable improvement, especially given the relatively low baseline values.

These results are in line with previous studies suggesting that 12 weeks of post-surgery exercise positively correlated with improvements in health-related quality of life [35,36]. Furthermore, the results provide additional evidence for the potential of increased fitness to contribute to extra improvements in depression after gastric bypass surgery. As the main implication, these results suggest that patients should participate in exercise training programs to increase their fitness status for optimal physical and mental outcomes of bariatric surgery.

Our results reinforce the existing evidence supporting the benefits of engaging in exercise after bariatric surgery [23,24,25,26,27,28,29,30] and contribute by addressing the potential of cardiopulmonary fitness in improving mental health following the surgery. Indeed, effective therapies to preserve wellbeing in severely obese people are needed. Recent studies have alerted about the limited effect of bariatric surgery to improve mental health compared to the non-surgical management of obesity [17,18,19,20,21]. This is further confounded by the pre-existing poor mental conditions among people with severe obesity [6,15]. In worst-case scenarios, bariatric patients who later commit suicide may not have a previous history of suicide attempts and/or depression [51]. In this context, efforts to improve the mental health of patients undergoing bariatric surgery are vital. Our findings suggest that interventions aimed to increase physical activity and fitness can play an important role in improving mental health after bariatric surgery [52]. Recent studies indicated that post-operative physical exercise is likely to reduce depression and anxiety symptoms [35,36]. Even though the results of these studies did not achieve statistical significance, the fact that relatively small changes in fitness can produce extra improvements in mental health is a promising finding. These findings support the well-documented benefits of the inclusion of exercise training in the clinical follow-up trajectory after bariatric surgery [31]. Arguably, the effect of exercise training will be maximized when combined with regularly conducted psychological therapies [34,53]. Likewise, in addition to individualized and gradual exercise and educational programs, encouraging lifestyle physical activity is paramount to achieving long-term results [54].

The use of accessible field-based tools to assess cardiopulmonary fitness, such as the 6MWT and accelerometry, enhances the practical applications of this study. The 6MWT has been shown as a tolerable [55] and highly reproducible fitness indicator among people with obesity [48]. Given the observed relationship between 6MWT performance and depression, it seems advisable to introduce this test as a clinical control tool for monitoring both physical and mental progress after bariatric surgery. Likewise, accelerometry is considered superior to self-administered questionnaires that fail to provide reliable estimates of physical activity levels among people following bariatric surgery, as they tend to overestimate physical activity compared to objective accelerometry [56]. This is important, since overestimations could lead to the prescription of exercise intensities, which are unachievable and may result in decreased adherence to exercise training sessions and, therefore, limited training effect [57].

This work has some important strengths, such as the length of follow-up and the use of practical, accessible, and reliable measures of physical activity and cardiopulmonary fitness. Additionally, to ensure a proper physical conditioning assessment and prescription, it seems pertinent to integrate sport science specialists to optimize the pre- and post-surgery process and conduct supervised and monitored interventions to improve the outcomes of the surgery [58,59]. This is particularly relevant given the limited adaptive response and effectiveness of general exercise guidelines compared to tailored and individualized exercise programs [60,61]. In general, our findings support the need for bariatric centers to provide complex care, including nutritional, behavioral, psychological, but also physical activity and exercise counseling.

This study also has certain limitations. The identified associations should be interpreted as exploratory given the non-randomized, non-blinded, observational nature of the study; thus, conclusions about the causal relationships are limited. Furthermore, the convenience sample of patients recruited from a single hospital may not fully represent the population of people who underwent bariatric surgery. Thus, future experimental studies are needed to confirm the effectiveness of particular exercise programs to improve mental health and the mediation role of cardiopulmonary fitness following bariatric surgery.

## 5. Conclusions

Increases in cardiopulmonary fitness were associated with greater improvements in depression score among patients undergoing bariatric surgery after controlling for age, sex, change in weight, change in the daily number of steps, and presence of comorbidities as covariates. These findings support patients’ engagement in physical exercise training programs after bariatric surgery to increase their activity levels and optimize the recovery outcomes, while improving mental health and wellbeing.

## Figures and Tables

**Table 1:** Baseline characteristics of patients prior to bariatric surgery (N = 26).

Characteristic	Value
Age (y)	45.4 ± 9.0
Sex (female/male)	20/6
Comorbidities (No. of patients)	
Type 2 diabetes	9
Hypertension	13
Sleep apnea	3
Mental illness (depression, anxiety)	9
Body composition	
Bodyweight (kg)	130.3 ± 21.9
BMI (kg·m^−2^)	45.1 ± 7.4
Body fat percentage (%)	43.8 ± 4.8
Cardiopulmonary fitness	
6MWT (m)	414 ± 59
Habitual physical activity	
Daily step count	5025 ± 2242 ^a^
Mental health (HADS)	
Anxiety	6.8 ± 3.4
Depression	6.2 ± 3.9
Fatigue (MAF)	
GFI	27.9 ± 10.3
Health-related quality of life (SF–36)	
Physical functioning	58.3 ± 24.5
Role—physical	51.9 ± 38.7
Bodily pain	63.6 ± 30.9
General health	60.6 ± 19.5
Vitality	51.5 ± 20.6
Social functioning	64.2 ± 29.4
Role—emotional	79.5 ± 32.8
Mental health	69.7 ± 16.9

^a^ The mean daily step count is based on data from 23 patients (88%) due to the insufficient number of valid days at baseline in 3 patients.

**Table 2:** Changes in body composition, mental health, and quality of life at 1, 3, and 6 months after surgery (N = 26).

Variable	1 Month after Surgery	3 Months after Surgery	6 Months after Surgery
Body composition			
Body weight (kg)	−13.8 (−15.8 to −11.8)	−26.1 (−30 to −22.2)	−35.3 (−39.9 to −30.7)
BMI (kg.m^−2^)	−4.8 (−5.5 to −4.1)	−9.1 (−10.3 to −7.8)	−12.4 (−14.1 to −10.8)
Body fat percentage (%)	−2.0 (−2.9 to −1.1)	−5.9 (−7.0 to −4.7)	−10.2 (−11.7 to −8.6)
Cardiopulmonary fitness			
6MWT (m)	not assessed	36.1 (24.5 to 47.6)	not assessed
Habitual physical activity			
Daily step count ^a^	not assessed	1260 (204 to 2315)	not assessed
Mental health (HADS)			
Anxiety	−1.8 (−2.9 to −0.7)	−3.0 (−4.2 to −1.8)	−3.9 (−5.1 to −2.7)
Depression	−2.5 (−3.6 to −1.4)	−3.1 (−4.3 to −1.9)	−3.0 (−4.2 to −1.7)
Fatigue (MAF)			
GFI	−4.2 (−6.6 to −1.8)	−10.9 (−14.8 to −7.1)	−13.2 (−17.2 to −9.3)
Health-related QoL (SF–36)			
Physical functioning	5.2 (−7.5 to 17.8)	20.8 (9.7 to 31.8)	24.4 (11.7 to 37.1)
Role—physical	32.7 (18 to 47.4)	26.0 (11.0 to 41.0)	31.7 (18.5 to 44.9)
Bodily pain	−2.2 (−17 to 12.5)	12.4 (−0.9 to 25.8)	19.4 (6.9 to 32.0)
General health	−2.3 (−10.1 to 5.5)	11.9 (4.2 to 19.6)	13.1 (4.2 to 21.9)
Vitality	3.5 (−4.8 to 11.7)	9.2 (2.1 to 16.3)	4.2 (−4.8 to 13.2)
Social functioning	0.9 (−9.3 to 11.0)	16.7 (4 to 29.4)	15.3 (3.3 to 27.3)
Role—emotional	−2.6 (−17.5 to 12.4)	12.8 (1.3 to 24.4)	5.2 (−6.7 to 17.0)
Mental health	4.6 (−2.4 to 11.6)	7.7 (0.6 to 14.8)	4.3 (−3.3 to 11.9)

Results are presented as changes from baseline assessment prior to surgery (95% confidence intervals). For the measures of mental health (range 0–21) and fatigue (range 1–50), the lower values (i.e., decreases) are considered desirable. For the quality-of-life measures (range 0–100), the greater values (i.e., increases) are considered desirable. HADS: Hospital Anxiety and Depression Scale, MAF: Multidimensional Assessment of Fatigue. SF–36: Short-Form Health Survey (QoL: Quality of life). GFI: Global Fatigue Index. ^a^ The mean change in daily step count is based on data from 19 patients due to the insufficient number of valid days at either baseline or three months after surgery in 7 patients.

**Table 3:** The effect of changes in distance walked during the 6MWT on improvements in mental health, fatigue, and quality of life.

Variable	3 Months after Surgery				6 Months after Surgery			
	Model 1		Model 2		Model 1		Model 2	
	*B* (SE)	*p*-Value	*B* (SE)	*p*-Value	*B* (SE)	*p*-Value	*B* (SE)	*p*-Value
Mental health (HADS)								
Anxiety	−0.01 (0.02)	0.509	−0.01 (0.02)	0.556	−0.02 (0.02)	0.410	−0.01 (0.02)	0.447
Depression	−0.05 (0.02)	0.005 *	−0.05 (0.02)	0.009 *	−0.06 (0.02)	0.007 *	−0.05 (0.02)	0.002 *
Fatigue (MAF)								
GFI	−0.04 (0.07)	0.527	−0.03 (0.10)	0.746	−0.03 (0.07)	0.644	−0.05 (0.09)	0.662
Health-related QoL (SF–36)								
Physical functioning	0.08 (0.19)	0.675	−0.08 (0.23)	0.746	0.26 (0.22)	0.250	−0.08 (0.25)	0.743
Role—physical	−0.06 (0.26)	0.810	−0.10 (0.38)	0.806	0.05 (0.23)	0.834	−0.15 (0.30)	0.626
Bodily pain	0.46 (0.22)	0.044	0.49 (0.23)	0.053	0.36 (0.21)	0.093	0.29 (0.23)	0.232
General health	0.12 (0.13)	0.398	−0.07 (0.13)	0.586	0.31 (0.14)	0.041	0.11 (0.14)	0.453
Vitality	0.23 (0.12)	0.054	0.07 (0.14)	0.626	0.31 (0.15)	0.045	0.12 (0.16)	0.447
Social functioning	0.38 (0.21)	0.085	0.24 (0.25)	0.338	0.49 (0.19)	0.015	0.25 (0.21)	0.269
Role—emotional	0.32 (0.19)	0.107	0.26 (0.25)	0.309	0.53 (0.18)	0.007	0.33 (0.21)	0.145
Mental health	0.02 (0.12)	0.860	−0.14 (0.16)	0.414	0.19 (0.13)	0.143	−0.05 (0.13)	0.723

Results are presented as beta coefficients (*B*) and standard errors (SE). Models are based on data from all 26 patients. QoL: Quality of life. Model 1 only included change in 6MWT distance as the explanatory variable. Model 2 also included age, sex, change in weight, and change in the daily number of steps as covariates. For the measures of mental health (range 0–21) and fatigue (range 1–50), the lower values (i.e., decreases) are considered desirable. For the quality-of-life measures (range 0–100), the greater values (i.e., increases) are considered desirable. * Significant effects.

## Data Availability

The data presented in this study are available on request from the corresponding author. The data are not publicly available due to privacy.
